# Influence of rutin and its combination with metformin on vascular functions in type 1 diabetes

**DOI:** 10.1038/s41598-023-39442-6

**Published:** 2023-08-01

**Authors:** Sheba R. David, Penny Pei Ni Lai, Jestin Chellian, Srikumar Chakravarthi, Rajan Rajabalaya

**Affiliations:** 1 School of Pharmacy, University of Wyoming, Laramie, WY 82071 USA; 2 School of Medicine, International Medical University, No. 126, Jalan Jalil Perkasa 19, Bukit Jalil, 57000 Kuala Lumpur, Malaysia; 3 School of Pharmacy, International Medical University, No. 126, Jalan Jalil Perkasa 19, Bukit Jalil, 57000 Kuala Lumpur, Malaysia; 4 SEGi University and Colleges, No. 9, Jalan Teknologi, Taman Sains Selangor, 47810 Kota Damansara, Selangor Malaysia; 5 PAPRSB Institute of Health Sciences, Universiti Brunei Darussalam, Jalan Tungku Link, Bandar Seri Begawan, BE1410 Brunei Darussalam

**Keywords:** Drug discovery, Diseases, Medical research

## Abstract

The present work examined the effect of oral administration of rutin and its combination with metformin, an antidiabetic drug on blood glucose, total cholesterol and triglycerides level and vascular function in streptozotocin (STZ) -induced diabetic rats. Male Sprague Dawley rats were rendered diabetic by a single intraperitoneal injection of STZ (50 mg/kg). Rutin and metformin were orally administered to diabetic rats at a dose of 100 mg/kg and 300 mg/kg body weight/day, respectively, for 4 weeks. Plasma analysis was conducted to determine changes in the plasma glucose and lipid levels. Rat aortic ring reactivity in response to endothelium-dependent (acetylcholine, ACh) and endothelium-independent (sodium nitroprusside, SNP) relaxants, and to the α1-adrenergic agonist phenylephrine (PE) were recorded. Histology of pancreas, liver and kidney were evaluated. In results, rutin and metformin alone and in combination has led to significant improvements in blood glucose, cholesterol and triglyceride levels compared to diabetic group. Diabetic aortic rings showed significantly greater contraction in response to PE, and less relaxation in response to ACh and SNP. Treatment with rutin and metformin in combination significantly reduced PE-induced contraction and increased ACh-induced and SNP-induced relaxation in diabetes when compared to rutin or metformin alone. Significant histological improvements were seen with combination therapy. In conclusion, rutin and metformin combination therapy has the most potentiality for restoring blood glucose and lipid level as well as vascular function.

## Introduction

In 2021, approximately 8.4 million people had Type 1 diabetes worldwide, and it is expected that this number will increase to 17.4 million by 2040^1^. Type 1 diabetes with hyperglycaemia, results from the lack of insulin being secreted from the pancreatic β cells, which are destructed by the immune system by the alteration of endothelial function through oxidative stress^2^. Evidences have shown that oxidative stress has been recognized as the key initiating factor the several pathways of hyperglycaemic damage^3^. The consequence of these pathways eventually leads to the increase of ROS, which further diminish NO production or enhance its degradation^4^. Recent meta-analysis showed that, children and adults with type 1 diabetes have signs of large vascular endothelial dysfunction which predisposes them to many vascular complications such as diabetic retinopathy, nephropathy, neuropathy and cardiovascular diseases^5^. Overall, this results in disability and reduced life expectancy, and tremendous healthcare costs on society^2^.

In past few decades, the research on therapeutic interventions for the treatment of type 1 diabetes has increased tremendously^2,6^. However, patients who depend on insulin are still looking for effective curative therapy to mitigate hyperglycaemia. Several studies have shown that, metformin has a greater potential in reducing diabetes-related endpoints as compared with other antidiabetic drugs such as chlorpropamide, glibenclamide or insulin^7^. Besides this, studies have documented the use of metformin with other hypoglycaemic agents such as insulin and sulphonylureas to improve overall diabetic profile in patients with type 1 diabetes^8–10^. Furthermore, metformin has been attributed to its direct vasculoprotective characteristics^7^.

Considering the adverse effects and lack of effectiveness with the current insulin therapy, there has been an increase in search of other potential oral hypoglycaemic or safer natural products for the prevention of type 1 diabetes. Rutin is a well-known flavonoid found abundantly in onions, apples, tea and red wine with many potential pharmacological properties including anti-diabetic and vasorelaxant effects^11^. It has been observed that rutin reduces oxidative stress which is predominantly involved in the pathogenesis of type 1 diabetes and its complications^12^.

In addition, the antioxidative properties of rutin have garnered attention due to its potential as an alternative treatment to effectively target in mitigating various metabolic and vascular dysfunctions^12^. Moreover, the combined effects of rutin and metformin in the context of diabetes management and vascular function have not been extensively investigated. Therefore, the purpose of this study was to assess the effects of oral administration of rutin, metformin, and their combination on blood glucose, total cholesterol and triglyceride levels, as well as vascular function in streptozotocin (STZ)-induced diabetic rats. We aimed to evaluate the potential synergistic effects of rutin and metformin in restoring metabolic parameters and improving vascular function. Histological evaluations of the pancreas, liver, and kidney were also conducted to assess histological improvements associated with the treatments. Understanding the impact of rutin, metformin, and their combination on blood glucose control, lipid profiles, and vascular function could provide valuable insights into their therapeutic potential for managing diabetes-related complications. Overall, this study provides a better understanding of rutin anti-diabetic effects when consumed individually as well as concomitantly with metformin.

## Results

### Effect of treatment on blood glucose levels

The mean blood glucose value was significantly (*p* < 0.01) lower in diabetic rats treated with rutin (16.1 ± 0.6 mmol/l) and metformin (14.5 ± 1.0 mmol/l) as compared with diabetic control group rats (26.6 ± 2.1 mmol/l). However, rutin and metformin combination therapy seemed to excel in the glucose lowering effect as compared to the individual treatments (11.5 ± 1.1 mmol/l, *p* < 0.01) (Table 1).

**Table 1:** Effect of treatment on plasma glucose, total cholesterol and triglyceride levels (mmol/l) in rats at the end of the treatment.

Blood tests (mmol/l)	Normal	Diabetic	Rutin	Metformin	Rutin + Metformin
Glucose	9.27 ± 0.26	26.63 ± 2.12^##^	16.13 ± 0.61^##^**	14.45 ± 1.01^##^**	11.55 ± 1.12^#^*^$^^
Total cholesterol	1.49 ± 0.45	3.00 ± 0.30^##^	1.48 ± 0.27**	2.23 ± 0.86	1.40 ± 0.43**
Triglycerides	1.33 ± 0.38	4.57 ± 2.48^##^	1.59 ± 0.50**	1.09 ± 0.29**	1.42 ± 0.41**

^#^Compared to normal control (*p* < 0.05).

^##^Compared to normal control (*p* < 0.01).

**Compared to diabetic control (*p* < 0.01).

^$$^Compared to rutin treated group (*p* < 0.01).

^^^Compared to metformin treated group (*p* < 0.05).

### Effect of treatment on blood lipid levels

The mean total cholesterol level was significantly higher (*p* < 0.01) in diabetic control group compared to normal control group. After treatment with rutin alone (1.48 ± 0.27 mmol/l) or in combination with metformin (1.40 ± 0.43 mmol/l), the total cholesterol level decreased significantly (*p* < 0.01) compared to the diabetic control (3.00 ± 0.30 mmol/l), to a level that was comparable to the normal control (1.49 ± 0.45 mmol/l). The total cholesterol levels in metformin treated group (2.23 ± 0.86 mmol/l) and diabetic control group remained unchanged (*p* > 0.05) (Table 1). STZ-induced diabetes (4.57 ± 2.5 mmol/l) produced a significant (*p* < 0.01) increase in total triglycerides level than normal control group (1.33 ± 0.4 mmol/l). But treatment with rutin (1.59 ± 0.5 mmol/l) or metformin (1.09 ± 0.2 mmol/l) and in combination (1.42 ± 0.4 mmol/l), decreased the total triglycerides level significantly (*p* < 0.01) when compared to the diabetic control group (4.57 ± 2.5 mmol/l) (Table 1).

### Effect of treatment on vascular function

#### Phenylephrine (PE) induced contraction

**Figure 1:**
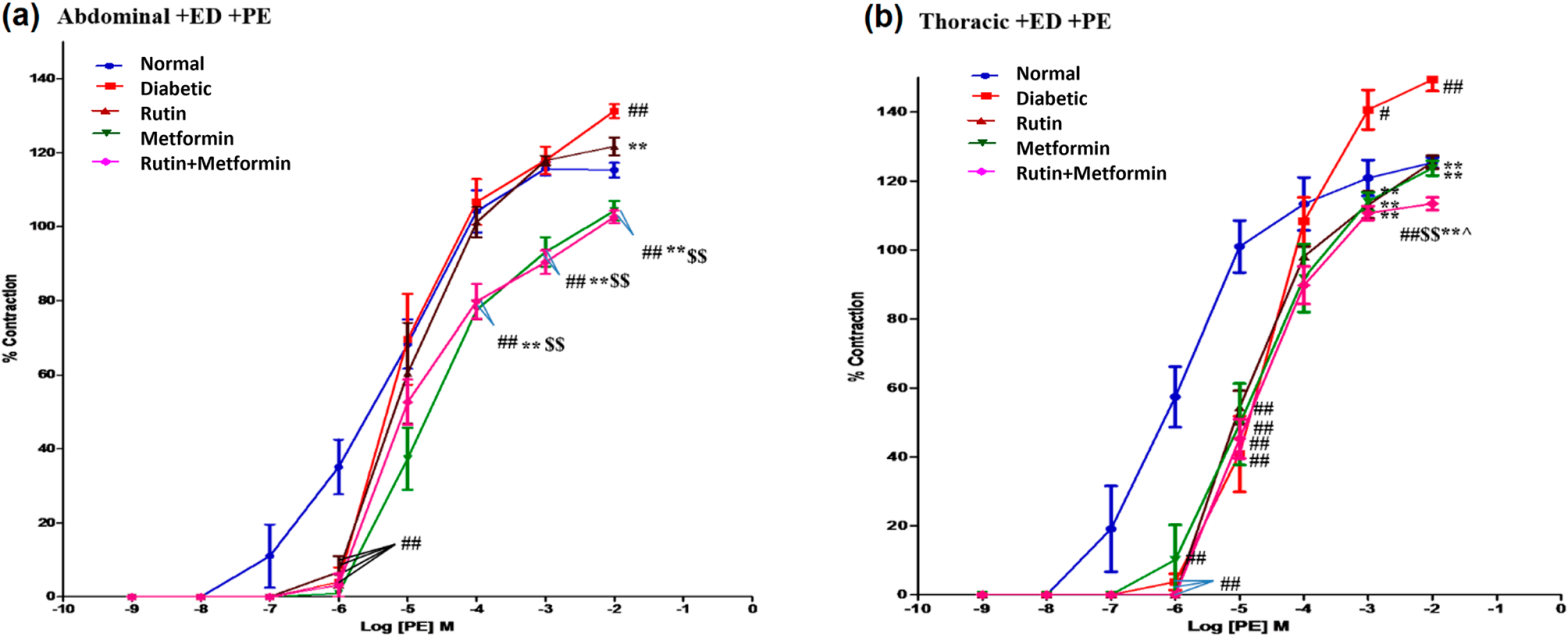
Cumulative concentration-effect curves showing contractile responses to α1 -adrenoceptor agonist Phenylephrine (PE) of endothelium intact (+ ED), (**a**) abdominal aortic rings, (**b**) thoracic aortic rings. Rutin (100 mg/kg), metformin (300 mg/kg). Symbols represent means ± SD. # denotes *p* < 0.05 versus response in vehicle-treated normal rat aortic rings; ## denotes *p* < 0.01 versus response in vehicle-treated normal rat aortic rings; ** denotes *p* < 0.01 versus response in vehicle-treated diabetic rat aortic rings; $$ denotes *p* < 0.01 versus response in rutin-treated diabetic rat aortic rings; ^ denotes *p* < 0.05 versus response in metformin-treated diabetic rat aortic rings.

The vasoconstriction of endothelium intact tissues with PE at the highest concentration tested (10^−2^) was significantly (*p* < 0.01) augmented in diabetic rat abdominal aortic rings compared with abdominal aortic rings taken from normal rats with (Fig. 1a). Treatment with rutin alone had demonstrated a significant (*p* < 0.01) reduction in the percentage of contraction at the highest concentration of PE as compared with diabetic aortic rings. However, treatment with metformin alone and together with rutin markedly reduced the responses to PE at 10^−4^, 10^−3^ and 10^−2^ concentration compared to rutin treated and normal and diabetic control (*p* < 0.01). Overall, diabetic rats treated with metformin and rutin exhibit the lowest amount of contraction in response to PE.

The vasoconstriction of PE at 10^−3^ and 10^−2^ concentration was significantly (*p* < 0.01) augmented in diabetic rat thoracic aortic rings as compared with thoracic aortic rings taken from normal rats (Fig. 1b). Treatment with rutin or metformin has demonstrated a significant (*p* < 0.01) reduction in the percentage of contraction at 2 of the highest concentration of PE as compared with diabetic aortic rings. However, treatment with metformin and rutin showed the most significant (*p* < 0.01) reduction in the response to PE at 10^−3^ and 10^−2^ concentration compared to the diabetic control. The maximal percentage contraction of the rutin metformin combination in response to PE 10^−2^ seems to be significantly (*p* < 0.01) more effective than the normal control. Besides this, the effect of combination therapy was also found to be significantly better at the highest concentration compared to the rutin treatment and metformin treatment alone.

**Figure 2:**
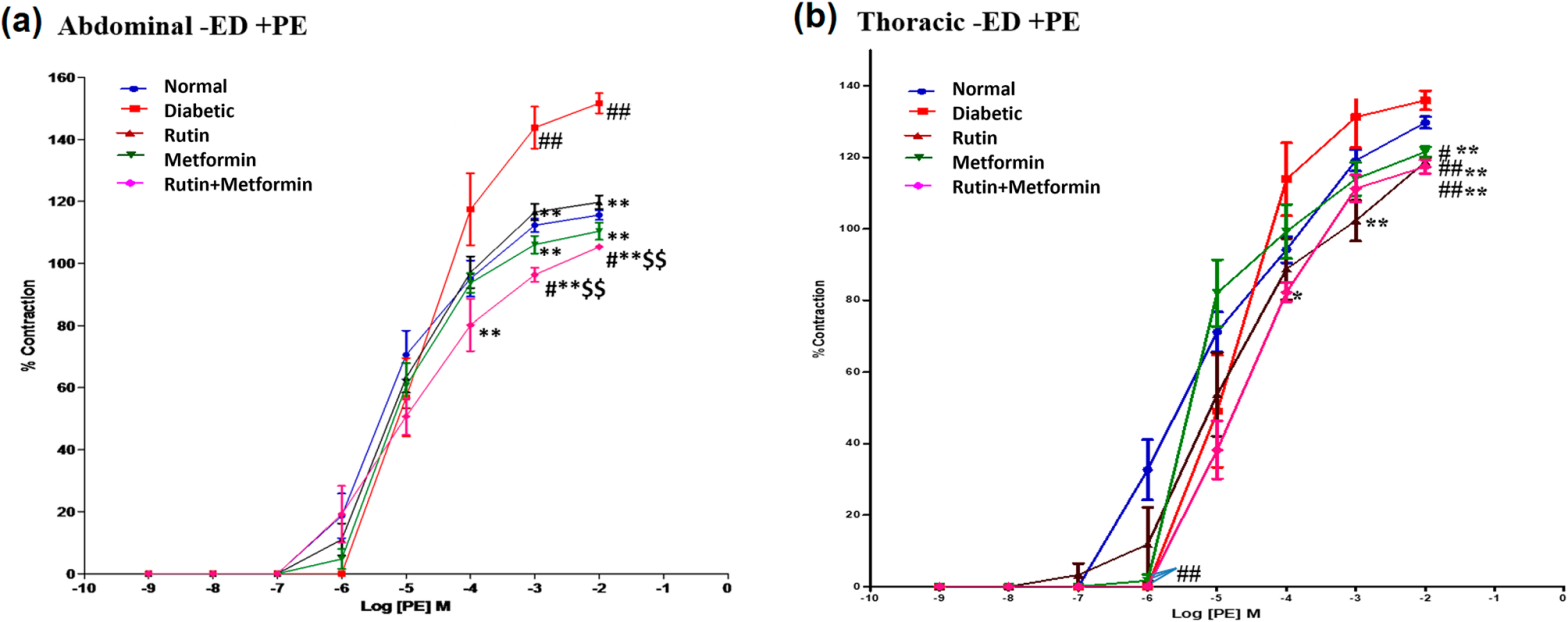
Cumulative concentration-effect curves showing contractile responses to α1 -adrenoceptor agonist Phenylephrine (PE) in endothelium-denuded (-ED), (**a**) abdominal aortic rings, (**b**) thoracic aortic rings. Rutin (100 mg/kg), metformin (300 mg/kg). Symbols represent means ± SD. # denotes *p* < 0.05 and ## denotes *p* < 0.01 versus response in vehicle-treated normal rat aortic rings; * denotes *p* < 0.05 and ** denotes *p* < 0.01 versus response in vehicle-treated diabetic rat aortic rings. $$ denotes *p* < 0.01 versus response in rutin-treated diabetic rat aortic rings.

Tissue contraction in response to PE in endothelium-denuded abdominal aortic rings from STZ-treated rats was significantly increased in comparison to normal rats at 10^−3^ and 10^−2^ concentration of PE (Fig. 2a). Aortic rings from those treated with rutin or metformin and in combination also showed a significant reduction in maximum contraction to PE (*p* < 0.01) but rutin and metformin in combination exhibited the greatest reduction in maximum contraction, to a level of PE-induced contraction that was significantly (*p* < 0.01) lesser than the normal control and rutin treated group.

Figure 2b shows the graph for the responses to PE of endothelium-denuded thoracic aorta of all treatment groups. Although diabetic aortic rings did not significantly enhanced tissue contraction in response to PE compared to the normal control. The aortic rings from those treated with rutin or metformin and in combination showed a significant (*p* < 0.05 and 0.01) reduction in maximum contraction to PE as compared to the diabetic and normal control. Also, rutin and metformin in combination exhibited the lowest percentage of contraction at 10^−2^ concentration of PE.

#### Acetylcholine (ACh) induced relaxation

**Figure 3:**
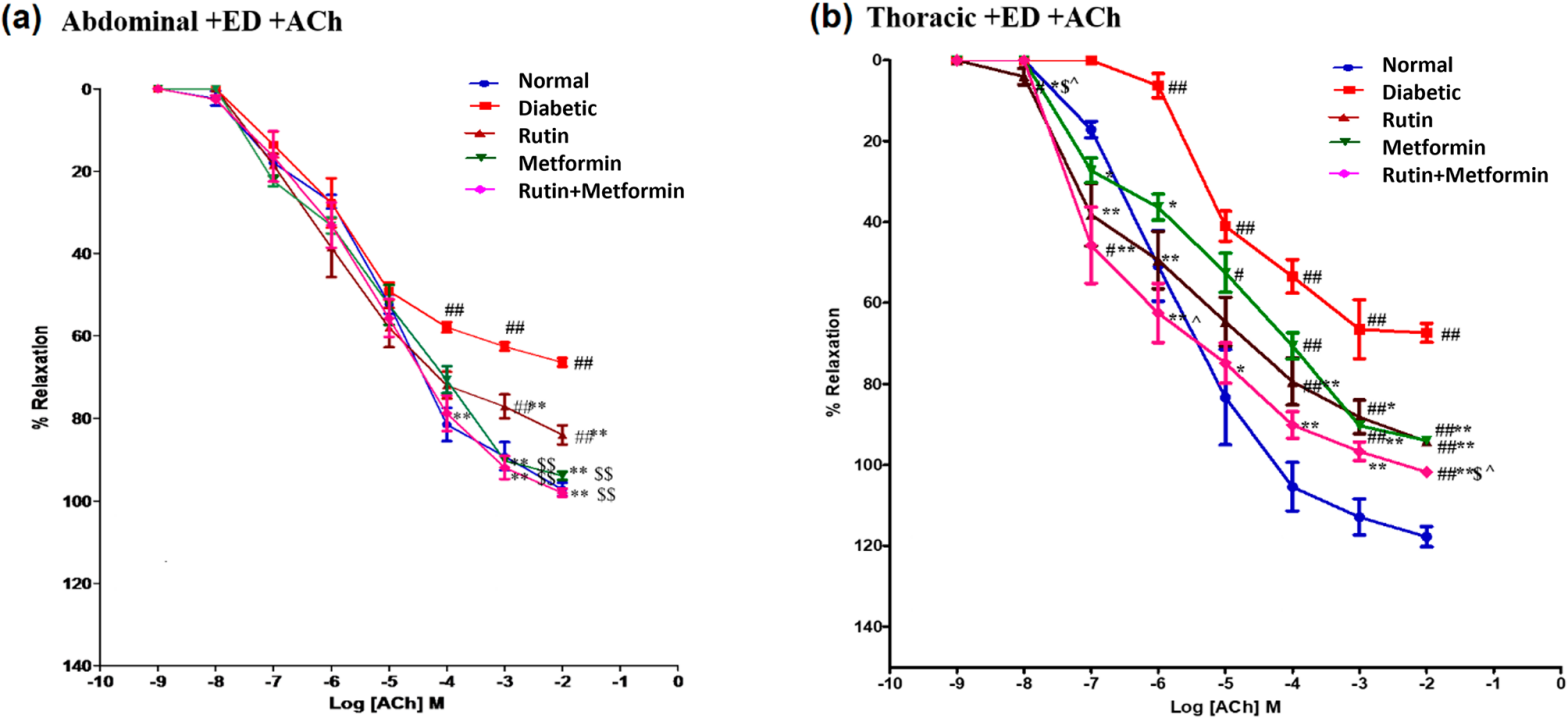
Cumulative concentration-effect curves showing endothelial dependent relaxation responses to Acetylcholine (ACh) in endothelium-intact (+ ED), (**a**) abdominal aortic rings, (**b**) thoracic aortic rings. Rutin (100 mg/kg), metformin (300 mg/kg). Symbols represent means ± SD. # denotes *p* < 0.05 and ## denotes *p* < 0.01 versus response in vehicle-treated normal rat aortic rings; * denotes *p* < 0.05 and ** denotes *p* < 0.01 versus response in vehicle-treated diabetic rat aortic rings; $$ denotes *p* < 0.01 and $ denotes *p* < 0.05 versus response in rutin-treated diabetic rat aortic rings; ^ denotes *p* < 0.05 verses response in metformin-treated diabetic rat aortic rings.

Aortic rings were pre-contracted with a sub-maximal concentration of PE (10 µM) and increasing concentrations of ACh (1 nM to 1 µM) were subsequently added to relax the pre-contracted aorta. Figure 3a shows the ACh-dose response curves of endothelium intact tissues from the abdominal aorta. From the curve, it can be understood that ACh-mediated relaxation was significantly reduced in diabetic rat aortic rings compared with non-diabetic aortic rings at 10^−4^, 10^−3^ and 10^−2^ concentration of ACh. Rutin treatment had a significant effect on the vasorelaxant responses to ACh at 10^−3^ and 10^−2^ concentration when compared to the diabetic control, but metformin treatment alone and in combination with rutin showed completely normalized the vasorelaxant effect at similar concentration of ACh when compared to diabetic and rutin treated groups (*p* < 0.01), denoting more potent effects.

ACh-dose response curves of endothelium intact tissues from the thoracic aorta showed that there was a rightward shift in the ACh concentration response curve in diabetic rat aortic rings, indicating that ACh-mediated relaxation was significantly reduced in diabetic rat aortic rings compared with non-diabetic aortic rings at most concentrations of ACh (Fig. 3b). The treatment with Rutin or metformin and in combination showed significant improvement in ACh-induced vasorelaxation at 10^−3^ and 10^−2^ concentration of ACh, but it still remained significantly higher than the normal control. However, rutin plus metformin treated group showed the most significant (*p* < 0.05) reduction in ACh-induced maximal relaxation at 10^−2^ concentration compared to rutin or metformin treatment groups. To compare, overall, rutin treated group showed a more depressed line curve with more significant (*p* < 0.01) amelioration of vasorelaxant responses to ACh at certain concentrations (10^−7^, 10^−6^, 10^−4^, 10^−3^) than metformin treated group, indicating that rutin had a greater vasorelaxant effect as compared to metformin, though non-significant. However, the line curve of rutin plus metformin treatment group was found to be more depressed than the rest except for the normal control, indicating that combination treatment caused the most significant correction of reduced ACh-induced relaxation in diabetic aortic at most concentrations, even though the maximal effect still remains higher than the normal control.

#### Sodium nitroprusside (SNP) induced relaxation

**Figure 4:**
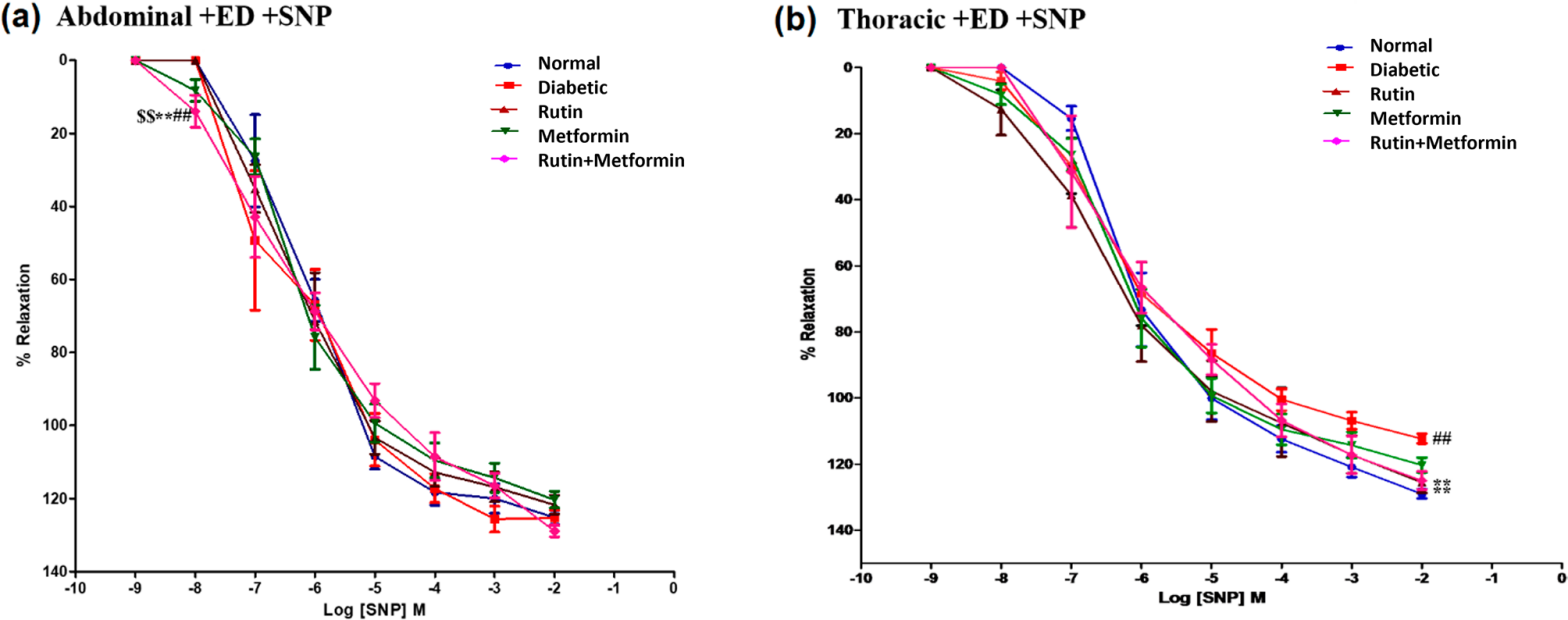
Cumulative concentration-effect curves showing endothelial independent relaxation responses to sodium nitroprusside (SNP) in endothelium-intact (+ ED), (**a**) abdominal aortic rings, (**b**) thoracic aortic rings. Rutin (100 mg/kg), metformin (300 mg/kg). Symbols represent means ± SD. ## denotes *p* < 0.01 versus response in vehicle-treated normal rat aortic rings; ** denotes *p* < 0.01 versus response in vehicle-treated diabetic rat aortic rings; $$ denotes *p* < 0.01 versus response in rutin-treated diabetic rat aortic rings.

Increasing concentrations of SNP (1 nM to 1 µM) were applied to the PE (10 µM) pre-contracted aortic rings to determine the SNP concentration response curve (Fig. 4a). The Tukey post hoc test showed no statistical difference between all the treatment groups in the abdominal aorta.

Figure 4b shows the result of relaxations induced by SNP in thoracic aortic rings from the various experimental groups. The relaxation curves for SNP for the various groups were similar, other than a significant reduction in the peak relaxation recorded for the control diabetic rings compared to the normal control aortic ring (*p* < 0.01) and significant improvements in the SNP-induced maximal relaxation were found for the rutin as well as rutin plus metformin group compared to the diabetic control aortic ring (*p* < 0.05).

**Figure 5:**
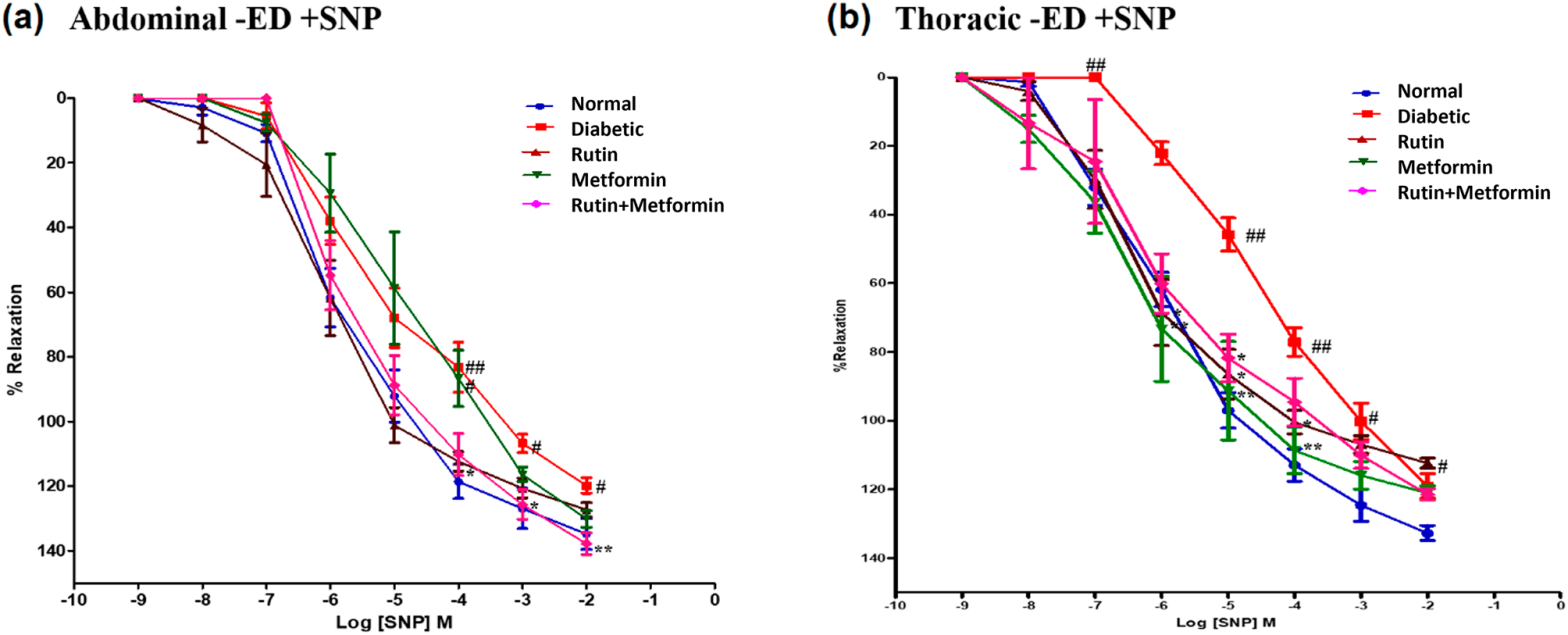
Cumulative concentration-effect curves showing endothelial independent relaxation responses to sodium nitroprusside (SNP) in endothelium-denuded (-ED), (**a**) abdominal aortic rings, (**b**) thoracic aortic rings. Rutin (100 mg/kg), metformin (300 mg/kg). Symbols represent means ± SD. # denotes *p* < 0.05 and ## denotes *p* < 0.01 versus response in vehicle-treated normal rat aortic rings; * denotes *p* < 0.05 and ** denotes *p* < 0.01 versus response in vehicle-treated diabetic rat aortic rings.

Diabetic endothelium denuded abdominal aortic rings showed significantly (*p* < 0.05 and 0.01) reduced endothelial-independent relaxation in SNP concentration of 10^−4^, 10^−3^ and 10^−2^ concentration (Fig. 5a). Treatment of diabetic rats with rutin plus metformin showed significantly (*p* < 0.05) improved response to SNP at concentration of 10^−3^ and 10^−2^ as compared to diabetic aortic rings. However, treatment with rutin and metformin individually did not significantly affect the aortic response to SNP.

Diabetic endothelium denuded thoracic aortic rings showed significantly (*p* < 0.05 and 0.01) reduced endothelial-independent relaxation in SNP concentrations of 10^−5^, 10^−4^ and 10^−3^ (Fig. 5b). Treatment of diabetic rats with rutin and metformin individually only showed significantly improved response to SNP at concentrations of 10^−5^ and 10^−4^ as compared to diabetic aortic rings. However, maximum relaxation at 10^−2^ concentration of SNP were enhanced in rutin treated group (*p* < 0.01).

### Histopathology

**Figure 6:**
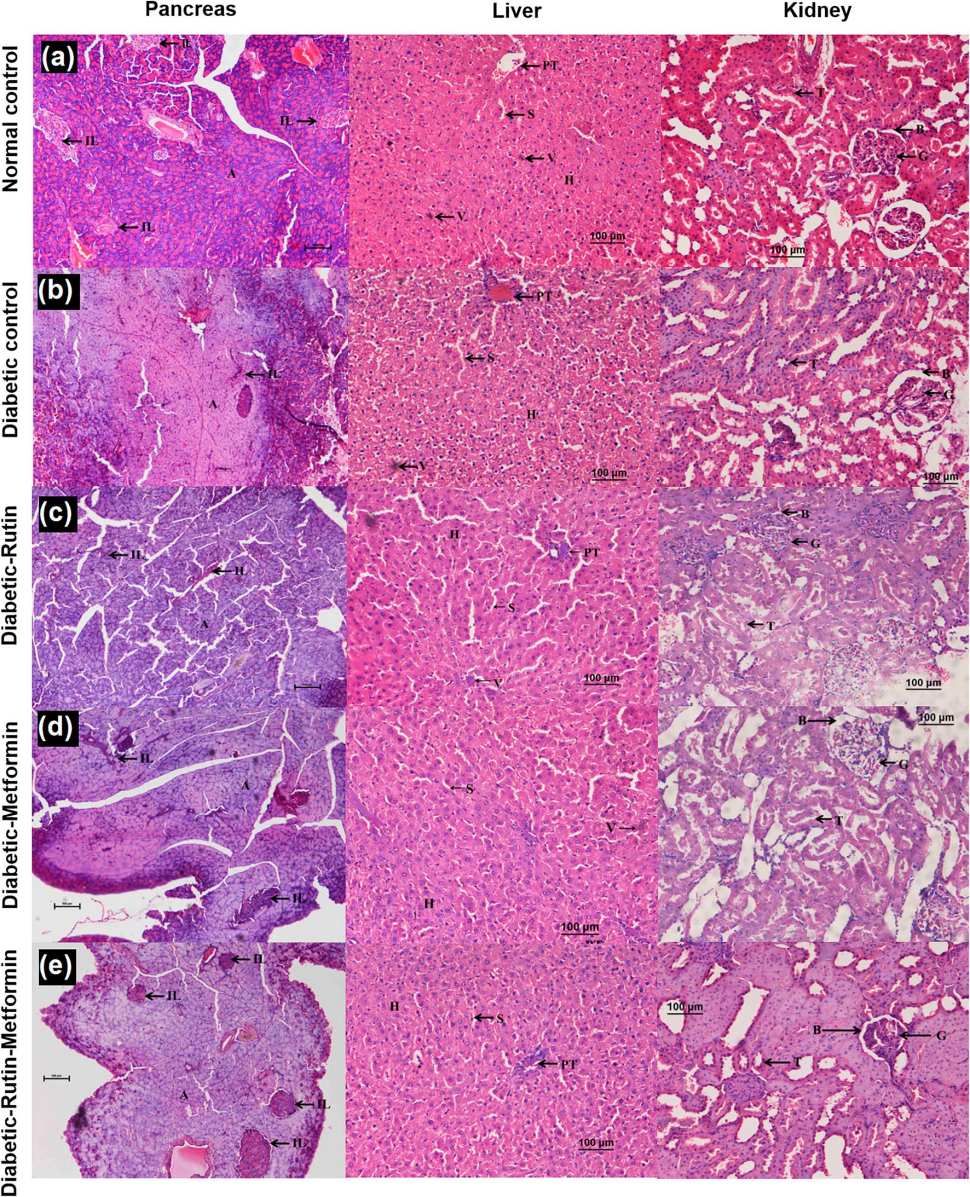
The effect of compounds on pancreas, liver and kidney. Rutin (100 mg/kg). Metformin (300 mg/kg). 100 × magnification. IL: Islets of Langerhans. A: Acinii. H: Hepatocytes. S: Sinusoids. V: Central Vein. PT: Portal Triad. B: Bowman’s space. G: Glomerulus. T: Renal Tubules.

The rats of normal control group showed normal architecture in all the three organs such as pancreas (normal exocrine acini and islets with adequate cellularity and distribution), liver (normal hepatocytes and sinusoids, normal central vein architecture along with well-spaced portal triads, no evidence of congestion) and kidneys (normal glomeruli, tubules and interstitium) (Fig. 6a). Whereas rats in diabetes control group showed damaged pancreas with diffused endocrine and exocrine systems. The acini appeared necrotic in focal areas and the endocrine islets showed reduction in size, cell number and distribution (Fig. 6b). The liver and kidneys appeared normal (Fig. 6b).

The rats fed with rutin showed signs of pancreas recovery as normal acini seen in the periphery of the exocrine areas (Fig. 6c). However, there were not much significant proliferation of the islets in terms of distribution, number and cellularity. The liver and kidney showed normal morphology. There was no congestion in the central vein or portal triads and glomeruli were normocellular (Fig. 6c). In metformin treatment group, pancreas showed few residual areas of damage admixed with abundant areas of regenerating acini and good distribution of islets with healthy cellularity (Fig. 6d). The liver and kidney showed normal architecture (Fig. 6d). The combination of rutin and metformin resulted in normal architecture of pancreas with endocrine islets (Fig. 6e). The distribution of islets was adequately spaced. The cellularity in the islets were good with some islets larger than the normal group. Liver and kidney showed normal architecture (Fig. 6e).

## Discussion

Plasma analyses revealed that the elevated glucose levels in diabetic rats were significantly reduced when supplemented with rutin or metformin and combination of both. These results corroborated well with the glucose lowering effects of metformin^13^ or rutin^14,15^. It has been reported that, the hypoglycaemic effect of rutin is believed to be mediated through, (a) enhancement of insulin secretion, (b) insulin binding affinity, (c) peripheral glucose consumption, (d) decreasing intestinal glucose and cholesterol absorption, and (e) diminishing activity of gluconeogenic and glycogenolytic enzymes^16^. Besides this, it was also proposed that the beneficial effect of rutin on glycaemic profile was due to its protective effect on pancreatic β-cells which preserved the insulin secretory capacity, thereby potentiating the secretion of insulin and glucose lowering effect^17^. Similarly, metformin is known to enhance glucose uptake in skeletal muscle and suppresses liver glucose secretion^18^. However, exact mechanism of glucose lowering is still unclear^2^. Overall, the combination of both metformin and rutin exhibited most significant blood glucose lowering effects in diabetic rats.

The lipid metabolism is commonly deranged in diabetic conditions due to lack of insulin to regulate the synthesis and breakdown of lipid in adipose tissues, thereby causing dyslipidaemia leading to atherosclerosis^19^. In view of the lipid profile, diabetic rats exhibited marked elevation of serum total cholesterol and total triglyceride level. The treatment of STZ diabetic rats with metformin significantly reduced total triglyceride, except cholesterol. These results are in congruity with Sin et al.^20^ who demonstrated significant improvement in triglyceride level following metformin treatment to type 2 diabetic patients. On the contrary, a systemic review and meta-analysis of randomized- controlled clinical trials suggested that metformin had no intrinsic effect on serum triglyceride level but significantly reduced total cholesterol in patients with type 2 diabetes^21^.

On the other hand, the rutin treatment in diabetic rats had more potent effect in decreasing total cholesterol and less effective in decreasing triglyceride as compared with metformin fed group. These results are in agreement with Ahmed et al.^16^, who demonstrated rutin mediated alleviation of serum total lipids, cholesterol, and triglycerides in diabetic rats. The ability of rutin to reduce cholesterol and triglycerides could be explained by the insulin releasing capacity, insulin binding affinity, decreasing intestinal cholesterol absorption and inactivation of hepatic HMG-CoA^16^. Overall, the treatment with rutin and metformin exerted the greatest hypolipidemic effect as compared with their individual counterparts.

This study demonstrated that after 8 weeks of diabetes, endothelial dysfunction was evident in the diabetic rat aorta. It was observed that, compared with normal rat aortic rings, α1-receptor agonist-contractions induced by PE were augmented in endothelial intact aortic rings from diabetic rats whereas endothelial-dependent relaxation induced by ACh were blunted in diabetic aortic rings. This data is consistent with earlier reports showing enhanced contractions in response to α1-adrenergic stimulant PE and reduced relaxation in response to the endothelial-dependent vasodilator ACh in vascular preparations from diabetic animals^22^. Multiple mechanisms have been implicated in the event of endothelial dysfunction in response to elevated (acute or prolonged) blood glucose levels, but increased oxidative stress seemed to be the first alteration triggering several others^23^. The deterioration of vascular function in diabetic aortic rings seen in this study could be attributed to the increased production of superoxide anions in the presence of hyperglycemia.

SNP-induced relaxation was not significantly reduced in endothelial intact diabetic rings except in the highest concentration of SNP in thoracic aorta. Whereas in the case of endothelium denuded vessels, SNP-induced relaxation was significantly reduced in both abdominal and thoracic aorta in various concentrations. This has been a rare finding as it has not been reported previously in diabetic subjects. Hence, the results of this study both agree and contrast with the results of previous vascular studies done in STZ-induced diabetic rats in which the response to SNP did not differ between normal and diabetics, although such difference was not excluded^24^. Sartoretto and co-workers^25^ have reported an impaired diabetic aortic response to both ACh and SNP, while Calver et al.^26^ showed that response to SNP was significantly less in the diabetics as compared to the healthy controls with no significant changes in the vascular response to ACh-induced relaxation. Therefore, the blunted vasodilator response to endothelial-independent reagents suggested that dysfunctional endothelium was not solely responsible for the impaired vascular relaxation observed in diabetic subjects. An impaired dilator response to SNP may be due to multiple abnormalities and some of them had been associated with smooth muscle dysfunction as a consequence of diabetes^27^. It has been postulated that such defect could be attributed to the decreased bioavailability of SNP/ NO or to the decreased sensitivity or responsiveness to NO.

In this study, we found that metformin, alone, reduced α1-adrenergic stimulant PE-induced contraction, increased ACh-induced endothelial-dependent and SNP-induced endothelial-independent relaxation in diabetic rat aortic rings. This finding is consistent with previous studies reporting that metformin elicited vasodilator effects in not only STZ-induced diabetic rats isolated aortas^25,28^ but also in humans^29^. Majithiya and co-workers^28^ found that the improvement in vascular function may be due to the involvement of NO pathway and cGMP as they have demonstrated that the enhanced ACh-induced relaxation in metformin-treated STZ-diabetic rats was blocked in the presence of N (gamma)-nitro-L-arginine methyl ester (L-NAME) and cGMP blocker but not in the presence of indomethacin. Indeed, studies investigating the aortic nitrite levels as an index of NO generation have shown that metformin treatment significantly increased the bioavailability of NO in aortas of STZ-diabetic rats and hence improved endothelial function^29^.

Treatment of diabetic rats with metformin also showed no significant improvement in endothelial-independent vasorelaxation in endothelium intact abdominal and thoracic aorta. Similar effects were also seen in both regions of the endothelial denuded aorta except for the thoracic aorta in moderate concentration of SNP. Thus, this is partially consistent with several studies who claimed a non-significant difference in the concentration-dependent relaxation induced by SNP in the treatment of metformin to diabetic aortic rings^28^.

Besides metformin, rutin treatment also attenuated PE-induced contractions in both endothelium intact and denuded aortic rings and increased ACh-induced relaxation in diabetic rat aortic rings with greater effects seen in the thoracic aortas compared to metformin. The vasorelaxant effect induced by rutin has been previously reported by Fusi et al^30^. It has been suggested that rutin seemed to be dependent on the NO/ guanylate cyclase pathway^31^. The preservation of NO activity may be due to rutin’s ability to preserve the integrity of endothelial cells as rutin was found to prevent endothelial toxicity and apoptosis by inhibiting intracellular oxidant accumulation^32^.

As for the case of endothelium denuded vessels, aortic constriction seen in response to PE was heightened in diabetic abdominal aorta. Treatment with rutin and metformin either individually or in combination both reduced the vasoconstrictor response to PE in high concentrations. This shows that diabetes can also cause dysfunction at the smooth muscle cell layer to produce ineffective control of vascular function^27^. Both rutin and metformin not only act on the endothelium but also at the smooth muscle cell layer to produce lower contractile response to PE. Similar effects can also be found in the thoracic aorta although with less noticeable effect.

As mentioned earlier, SNP-induced relaxation in both endothelium denuded abdominal and thoracic aortic rings showed that diabetic tissue exerts significantly less vasorelaxation compared to the normal control at certain concentration, but rutin plus metformin treatment significantly reversed that effect while rutin and metformin individual treatments did not show any significant difference in response in the abdominal aorta but significant reduction of SNP-induced relaxation was found in abdominal aorta; thus, ascertaining their synergistic effect.

In the assessment of vascular function, although rutin and metformin monotherapies showed improvement from the deterioration of vascular function in diabetes but combination therapy of both drugs exhibited greatest advancement in the result of the vascular experiment. To the best of our knowledge, this is the first report showing the beneficial effects of rutin and metformin in combination on vascular function in diabetes. Rutin and metformin combination therapy showed significantly stronger vasorelaxant effect than the monotherapy as it had not only attenuated PE-induced contractions in diabetic rings, but also enhanced relaxation to ACh as well as SNP. However, mechanism of action for this marked improvement of vascular function in combination group remains to be established. Reasons for this synergistic effect of rutin and metformin therapy may be related to the potent beneficial effects of both rutin and metformin individually and the fact that both of them share similar pathways which is the NO/guanylate cyclase pathway to improve endothelial function. However, there is room for the possibility of other pathways which may act similarly or separately between the two that coincide to bring about an overall synergistic effect on the vascular function. Furthermore, the results obtained with histological analysis are concurrent with the other results of this study.

In conclusion, the present study demonstrates that the combination treatment of rutin and metformin at the concentration of rutin 100 mg/kg and metformin 300 mg/kg seemed to be a good dosage with favourable effects on both the biochemical profile as well as vascular function. These results raise the potentiality of rutin and metformin combination as valuable treatments for improving the vascular function in type 1 diabetes.

## Methods

### Materials

Rutin hydrate, phenylephrine hydrochloride (PE), acetylcholine (ACh) chloride, sodium nitroprusside (SNP), streptozotocin (STZ), metformin and Krebs salts were purchased from Sigma Chemicals Company (St. Louis, MO, USA).

### Animal studies

Thirty Male Sprague–Dawley rats aged between 10 and 14 weeks with body weight ranging from 150 to 200 g were obtained from University Putra Malaysia (UPM). All the animals were housed in the departmental animal holding facility in International Medical University (IMU) under controlled conditions (21–24 °C with 12 h light and 12 h dark cycles). The animals had free access to standard rat chow and reverse osmosis water throughout the experiment except when noted. Approval for the studies was obtained from the Joint Committee of Ethics, IMU, Kuala Lumpur, Malaysia with the approval no.: BMS/I-02/2012(01). The animal experiments and procedures were carried out according to the guidelines of ethical care and standard regulations. We have conducted the experiments in accordance with ARRIVE guidelines. All experimental animals were euthanized by cervical dislocation method which is one of the common methods for euthanasia.

After one week of acclimatization, the rats were randomly divided into euglycemic and diabetic groups. The diabetic group rats were injected with a single intraperitoneal dose of STZ (50 mg/kg body weight) dissolved in cold saline to induce the disease^33–36^. Plasmatic glycemia was examined 10 days after diabetes induction and the rats were considered as diabetics only if their blood glucose level exceeds 17 mmol/l. The rats were then maintained at the specified conditions for 4 weeks. Later, the diabetic and euglycemic rats were randomized into 5 groups (6 rats per group) as described below. Group 1: Normal control rats- 1% (w/v) carboxymethyl cellulose (Sigma-Aldrich, Malaysia); Group 2: Diabetic control rats- vehicle; Group 3: Diabetic + Rutin (100 mg/kg^14^); Group 4: Diabetic + Metformin (300 mg/kg^37^); Group 5: Diabetic + Rutin (100 mg/kg) + Metformin (300 mg/kg); All treatments were given daily once for 4 weeks using the oral gavage needle.

### Biochemical analysis

Blood collected from tail artery was placed in heparinised vacutainers, centrifuged and separated plasma was stored at -80 °C until analyses. Total blood glucose, cholesterol and triglyceride levels were evaluated using a Dimension Xpand Plus analyser (Siemens, USA).

### Vascular function test

At end of the trial, the rats were euthanized by cervical dislocation. The descending thoracic and abdominal aorta from various treatments and control group rats were excised and adjoining fat and connective tissues were removed as per the method described by Boutouyrie et al.^38^ Appropriate care was taken to preserve the integrity of the endothelium. The vessels were neither stretched nor were the luminal surfaces of the rings disturbed. The aorta was then cut into small rings (3–5 mm in width) and suspended between two wire stirrups in a jacketed organ bath containing 7 ml of normal Krebs physiological solution (KPS), whose composition is NaCl 118.2, KCl 4.7, CaCl_2_ 2H_2_O 2.5, KH_2_PO_4_ 1.2, MgCl_2_ 1.2, glucose 11.7, NaHCO_3_ 25.0, and EDTA 0.026 mM. The bath solution was maintained at 37 °C and oxygenated continuously with a mixture of 95% O_2_ and 5% CO_2_. The rings were then progressively stretched to a preload tension of 1.5 g and allowed to equilibrate for 45 min.

During this period, the bath solution was replaced every 15 min. Following the equilibration period, aortic rings were exposed thrice (5 min each) to 399 µl isotonic 80 mM potassium chloride solution (high KCl). After washout of responses to high KCl, the relaxation responses to ACh and SNP or contractile responses to PE were recorded in the aortic rings^22^ using the Power Lab recording system (AD Instruments, Australia).

To access the integrity of the endothelium, the aortas were pre-contracted with a dose of PE (10 µM) until it reached a peak within 5 min, followed by a dose of ACh (10 µM) to relax the artery rings. If ACh-induced relaxation was greater than 50% of the pre-contracted tone, the endothelium was considered to be functionally intact while endothelium denuded rings were required to exhibit less than 5% relaxation to ACh^22^.

### Contractile responses to PE

The contractile responses of the aortic rings to cumulatively increasing concentrations of PE (1 nM to 1 µM) were recorded at intervals of 4 min.

### Relaxations to ACh and SNP

The relaxation to ACh and SNP in rat aorta was examined in PE pre-contracted aortic rings. The rings were exposed to single concentration of PE (10 µM) and at the peak of the contraction the relaxations to cumulatively increasing concentrations of ACh (1 nM to 1 µM) or SNP (1 nM to 1 µM) were recorded at 4 min intervals.

### Histological analysis

At end of the treatment, pancreas, liver and kidney were removed from sacrificed animals and stored in 10% (v/v) formalin. The tissues were then processed, blocked, sliced and kept on microscopic slides for haematoxylin and eosin (HE) staining. The Nikon 8.1 mega-pixel microscope ECLIPSE TS100 was used to capture the histological photomicrographs.

### Statistical analysis

All the data was expressed as means ± standard deviation (SD). The concentration–response curves of aortic rings to PE, ACh, and SNP for each experimental condition was plotted using non-linear regression software (Prism version 5.0 Graph pad software USA). The observed responses were analyzed for statistical significance the one-way analysis of variance (ANOVA) for multiple value comparison, followed by appropriate (Tukeys) post hoc test (Prism 2.0, GraphPad Software, USA). Same statistical methods were used for the calculation of statistical differences in plasma analyses results. A value of *p* < 0.05 was considered statistically significant.

## Data Availability

The datasets generated during and/or analysed during the current study are available from the corresponding author on reasonable request.
